# Maternal—Fetal rejection reactions are unconstrained in preeclamptic women

**DOI:** 10.1371/journal.pone.0188250

**Published:** 2017-11-27

**Authors:** Tina A. Nguyen, Daniel A. Kahn, Andrea I. Loewendorf

**Affiliations:** Department of Obstetrics and Gynecology, Division of Maternal-Fetal Medicine, David Geffen School of Medicine, UCLA, Los Angeles, California, United States of America; Jackson Laboratory, UNITED STATES

## Abstract

The risk factors for preeclampsia, extremes of maternal age, changing paternity, concomitant maternal autoimmunity, and/or birth intervals greater than 5 years, suggest an underlying immunopathology. We used peripheral blood and lymphocytes from the UteroPlacental Interface (UPI) of 3^rd^ trimester healthy pregnant women in multicolor flow cytometry—and *in vitro* suppression assays. The major end-point was the characterization of activation markers, and potential effector functions of different CD4—and CD8 subsets as well as T regulatory cells (Treg). We observed a significant shift of peripheral CD4 –and CD8- T cells from naïve to memory phenotype in preeclamptic women compared to healthy pregnant women consistent with long-standing immune activation. While the proportions of the highly suppressive Cytokine and Activated Treg were increased in preeclampsia, Treg tolerance toward fetal antigens was dysfunctional. Thus, our observations indicate a long-standing inflammatory derangement driving immune activation in preeclampsia; in how far the Treg dysfunction is caused by/causes this immune activation in preeclampsia will be the object of future studies.

## Introduction

Preeclampsia is a mysterious condition that affects 3–17% of pregnancies worldwide[1]. Undoubtedly, the reader’s life in some way has been touched by preecampsia. The mother and fetus may suffer serious complications including hypertension, organ failure, progression to seizures (eclampsia), prematurity, and death[1]. Currently, the diagnosis relies on serial blood pressure and proteinuria monitoring over a 24-hour period. The only effective treatment is delivery. A molecular explanation for preeclampsia that could guide more robust treatments is a major unmet medical need. Risk factors for preeclampsia include extremes of maternal age, changing paternity, concomitant maternal autoimmunity, and/or birth intervals greater than 5 years all suggesting involvement of immunologic mechanisms[1]. The pathology of preeclampsia has been investigated at many levels including placentation abnormalities and novel molecular descriptions of the hypertensive phenotype [2–4]. Fetal-maternal immune alterations are likely the initiating factors of this cascade of events as suggested by the risk factors and the unique immunologic setting of pregnancy.

Prior considerations of the immunopathology of preeclampsia have focused on individual components of a potential immune derangement such as changes in serum inflammatory cytokines [5]. We report here phenotypic and functional parameters of T cells and Treg in the periphery (peripheral blood lymphocytes, PBL) and at the uteroplacental interface (UPI) impacted by preeclampsia. In addition, we detail a disruption of functional Treg-mediated maternal tolerance to fetal antigen in preeclampsia. Taken together, the data related here provide a framework for understanding immunologic underpinnings of preeclampsia.

## Materials and methods

### Ethics statement

Human subjects were recruited for participation after IRB approval (University of California, Los Angeles, Office of Human Research Protection Program, Medical IRB Committee-1 #11–003962). Each subject provided written informed consent prior to enrollment.

### Human subjects

Healthy women with and without a clinical diagnosis of preeclampsia in the third trimester with singleton pregnancies were recruited for participation between March 2009 and July 2014. Demographic and obstetrical characteristics from the recruited population are provided in Tables 1–3. White Blood Cell (WBC) count was obtained from review of the medical record for values obtained at admission to Labor and Delivery.

**Table 1:** Subject demographics for immunophenotype. Age, gravidity, and parity differences analyzed with a two-way Student’s T test. Ethnicity and advanced maternal age differences analyzed using a Fisher’s exact test.

Age	Healthy	Preeclampsia	
Range	19–42	22–40	p = 0.2473
Average	33.1	31.13	
Median	32.5	33.5	
Ethnicity			
Caucasian	59% (17)	38% (6)	p < 0.0001
Hispanic	28% (8)	19% (3)	
Black	10% (3)	19% (3)	
Asian	3% (2)	25% (4)	
Gravidity	2.73	1.6	p < 0.05
Parity	1.06	0.2	p < 0.05
Smoker	3% (1)	0%	
Comorbidities			
Advanced maternal age	41% (12)	38%(6)	p = 0.5419

**Table 2:** Subject obstetrical characteristics for immunophenotype. Mode of delivery differences analyzed using a Fisher’s exact test. Fetal birth weight and APGAR score differences analyzed using a two-way student’s T test.

Mode of Delivery	Healthy	Preeclampsia	
Vaginal	10% (3)	44% (7)	p = 0.013
Cesarean	90%(27)	56% (9)	
Fetal			
Average birth weight (g)	3490 ± 84g	3231 ± 191g	p = 0.2149
APGAR-1	8.40 ± 0.12	7.69 ± 0.44	p = 0.0524
APGAR-5	8.93 ± 0.05	8.81 ± 0.12	p = 0.3081

**Table 3:** Subject characteristics for Treg tolerance defect in preeclampsia. Age, gravidity, parity, and gestational age differences analyzed with a two-way student’s T test. Mode of delivery differences analyzed using a Fisher’s exact test.

	Healthy	Preeclampsia	
Age	28.8 ±2.1	30.8 ± 2.6	p = 0.5591
Gravidity	2.5 ± 0.6	1.8 ± 0.2	p = 0.2870
Parity	0.8 ± 0.3	1.8 ± 0.3	p = 0.0398
Fetal			
Gestational age (weeks)	39.1 ± 0.2	38.6 ± 0.6	p = 0.3919
Mode of delivery			
Vaginal	38% (3)	50% (3)	p = 0.0134
Cesarean	62% (5)	50% (3)	

### Tissue collection

Peripheral blood (5–20 ml) was collected into EDTA-containing tubes using standard aseptic venipuncture technique, usually in conjunction with placement of an IV line. Cells from the uteroplacental interface were obtained as follows: At the time of cesarean section, after delivery of the fetus and placenta, the hysterotomy was wiped clean of blood. Then, a sterile surgical sponge was used to wipe the intrauterine cavity, a standard procedure that ensures complete removal of the placenta. This surgical sponge was placed into a container with ~50ml of sterile PBS.

### Lymphocyte purification

Granulocytes were depleted utilizing the RosetteSep Granulocyte Depletion Cocktail (Stemcell Technologies, Vancouver, Canada) following manufacturer’s recommendations. Approximately 50 ml PBS wash of uteroplacental swipe were treated with 250μl of Cocktail. Granulocyte-depleted mononuclear cells were isolated by gradient centrifugation over Ficoll-Paque PLUS from GE Healthcare (Uppsala, Sweden) following manufacturer’s recommendations. Cells were washed twice with sterile PBS and enumerated utilizing an Accuri flow cytometer with Propidium Iodide exclusion of dead cells.

### *In vitro* suppression assay

Maternal and cord blood lymphocytes were isolated by gradient centrifugation over Ficoll-Paque PLUS from GE Healthcare following manufacturer’s recommendations. Cells were washed twice with sterile PBS and enumerated. A portion of maternal lymphocytes were anti-CD25-PE labeled followed by anti-PE-magnetic beads following manufacturers instructions (Miltenyi, San Diego, CA). CD25+ depleted cells were assessed for FoxP3 expression and similar depletion efficiencies were observed between healthy pregnant and preeclamptic pregnant women’s samples. Cord blood lymphocytes were exposed to 5000 Rads in a Cs^132^ irradiator. 200,000 maternal lymphocytes per well were incubated in a 96-well round bottom plate (BD-Falcon, San Diego, CA) with 200,000 irradiated cord blood lymphocytes as indicated for 120 hours in RPMI 1640 supplemented with 10% (vol/vol) heat-inactivated, 5.5 × 10^−5^ M ß-mercaptoethanol, 100 units/mL penicillin, 100 units/mL streptomycin, and 2.5 mM L-glutamine. Cells were cultured at 37°C with 5% CO_2_ in a humidified incubator. 1μCi of ^3^[H] Thymidine was included for the final 18–22 hours of the culture.

### *In vitro* re-stimulation assay for cytokine production

Approximately 5x10e5 purified lymphocytes were seeded in 200 μl RPMI1640 supplemented with 5% human AB serum (Gemini Bioproducts, West Sacramento, CA) and 5.5 × 10^−5^ M ß-mercaptoethanol, 100 units/mL penicillin, 100 units/mL streptomycin, and 2.5 mM L-glutamine. NK cell panels were incubated either without stimulation or with stimulation while T cells were always cultured in the presence of stimulation with 25ng/ml phorbol 12-myristate 13-acetate (PMA), 250ng/ml Ionomycin and BD Golgistop according to manufacturer’s recommendations (Becton Dickinson, Franklin Lakes, NJ) for 4.5 h. Cells were washed in PBS, stained with viability staining and surface antibodies and fixed in 1% Formalin/PBS/2%FCS overnight. Intracellular staining with antibodies or appropriate isotype controls was performed the next day using the BD Cytofix/Cytoperm kit according to manufacturer’s instructions (Becton Dickinson). Samples were included in the results when isotype control staining background was <1%.

### Phenotypic analysis via multicolor flow cytometry

Immediately after cell purification, 1x10^6^ cells were stained with Fixable Viability Dye eF780 (eBioscience) in 200 μl PBS in a 96 well u-bottom plate. The cells were pelleted and supernatant removed. Antibodies against surface antigens were added in 100 μl PBS and 1% FBS at the optimal concentrations determined by previous titration (Table 4) and incubated for 15 minutes at room temperature in the dark. Following two washes with PBS and 1% FBS, intra-nuclear FoxP3 staining was performed utilizing the FoxP3/Transcription Factor Staining Kit (eBioscience, San Diego, CA) per manufacturer’s instructions including the recommended 15min incubation with mouse serum prior to intra-nuclear staining. Cells were re-suspended in PBS and 1% FBS, transferred to FACS tubes and analyzed within 6 hours. Either isotype staining or Fluorescence Minus One (FMO) stains were performed. For isotype control staining, the appropriate antibody isotype or, if unavailable, a similar isotype coupled to the same fluorophore was used at the same concentration as the antibody. Histogram gating was performed using the isotype staining as a guide to set the gates. For FMO controls, all antibodies minus one (FoxP3) were stained [6]. Analysis was performed on a BD SORP LSR II analytic flow cytometer. Flow cytometer voltage settings were first tested for each new combination of antibodies and confirmed at the time of analysis using single-stained cells for each flurophore treated in the same fashion as the test samples (f.e. FoxP3-buffer treated) and following these general guidelines: voltages for fluorophores excited by the Yellow-Green laser (561nm) are set similar to each other with the exception of intranuclear FoxP3-PE; FoxP3-PE voltage is established using cells with a single stain of intranuclear FoxP3-PE; if several fluorophores excited by the violet laser (405nm) were combined in one panel, ascending voltages were used according to the fluorophore emission (Brilliant Violet 785> Brilliant Violet 711> Brilliant Violet 655 etc.). Post-acquisition analysis was performed with FlowJo (Treestar, Palo Alto, CA) with special attention to batch analysis: we found staining intensities of the same populations to vary between peripheral blood and cells of the uteroplacental interface hence re-gating was necessary when these types of samples were acquired together (for example CD56).

**Table 4:** Antibodies used for antigen detection. Intracellular/intranuclear antigens are noted in *italics*.

Antigen	Source	Clone	ng/test	Fluorochrome	Excitation	Longpass Dichroic Mirror	Bandpass filter
*Perforin A*	Biolegend	B-D48	500	Pacific Blue	405nm	blank	450/50
CD45RO	Biolegend	UCHL1	2500	Brilliant violet 421 or Pacific Blue	405nm	blank	450/50
CD4	Biolegend	OKT4	150	Brilliant violet 510	405nm	505LP	525/50
CD31	Biolegend	WM59	250	Brilliant violet 605	405nm	595LP	605/40
CD25	Biolegend	BC96	250	Brilliant violet 605	405nm	595LP	605/40
CD3	Biolegend	OKT3	250	Brilliant violet 605	405nm	595LP	605/40
CD45RA	Biolegend	HI100	125	Brilliant violet 605	405nm	635LP	660/40
CD69	Biolegend	FN50	250	Brilliant violet 655	405nm	635LP	660/40
CD45RA	Biolegend	HI100	125	Brilliant violet 655	405nm	635LP	660/40
CD4	Biolegend	OKT4	300	Brilliant violet 711	405nm	685LP	710/50
HLA-DR	Biolegend	L243	180	Brilliant violet 711	405nm	685LP	710/50
CD45RO	Biolegend	UCHL1	500	Brilliant violet 785	405nm	750LP	780/60
CD45RA	Biolegend	HI100	400	AF488	488nm	505LP	530/30
CD3	Biolegend	HIT3a	3000	AF488	488nm	505LP	530/30
*IFNγ*	Biolegend	4S.B3	250	AF488	488nm	505LP	530/30
CD3	eBioscience	UCHT1	500	PerCP-Cy5.5	488nm	685LP	695/40
*Granzyme A*	Biolegend	CB9	250	PerCP-Cy5.5	488nm	685LP	695/40
*IFNγ*	Biolegend	4S.B3	300	PerCP-Cy5.5	488nm	685LP	695/40
IL-2Rα	Biolegend	BC96	2500	AF700	640nm	685LP	710/50
CD27	Biolegend	O323	2500	AF700	640nm	685LP	710/50
CD8	Biolegend	HIT8a	2500	AF700	640nm	685LP	710/50
CD3	Biolegend	HIT3a	2500	AF700	640nm	685LP	710/50
*TNFα*	Biolegend	MAB11	500	AF700	640nm	685LP	710/50
Viability marker	eBioscience	Cat# 65–0865	1:1000	eF780	640nm	755LP	780/60
*IFNγ*	Biolegend	4S.B3	20	AF647	640nm	blank	670/30
CD4	Biolegend	OKT4	500	AF647	640nm	blank	670/30
CD28	Biolegend	CD28.2	500	APC	640nm	blank	670/30
*FoxP3*	BD	259D/C7	500	PE	561nm	blank	582/15
*IL-2*	Biolegend	MQH1-17H12	125	PE	561nm	blank	582/15
IL18Rα	Biolegend	H44	500	PE	561nm	blank	582/15
CCR7	BD	150503	500	PE-CF594	561nm	600LP	610/20
CD3	BD	UCHT1		PE-CF594	561nm	600LP	610/20
CD27	Biolegend	O323	500	PE/Cy7	561nm	750LP	780/60
HLA-DR	Biolegend	L243	125	PE/Cy7	561nm	750LP	780/60
*TNFα*	Biolegend	MAB11	1000	PE/Cy7	561nm	750LP	780/60
CD8	BD	RPA-T8	5 μl	BUV395	355nm	blank	
CD3	BD	UCHT1	5 μl	BUV395	355nm	blank	

### Statistical analysis

Differences in normally distributed populations were statistically analyzed using unpaired Student’s T test or analysis of variance (ANOVA) with Bonferroni post-test. Optimum clinical performance was determined using a Receiver Operator Curve (ROC) analysis. Statistical analyses were accomplished with PRISM (Graphpad, La Jolla, CA).

## Results

### The CD4 memory T cell compartment is significantly expanded in preeclamptic patients

**Fig 1:**
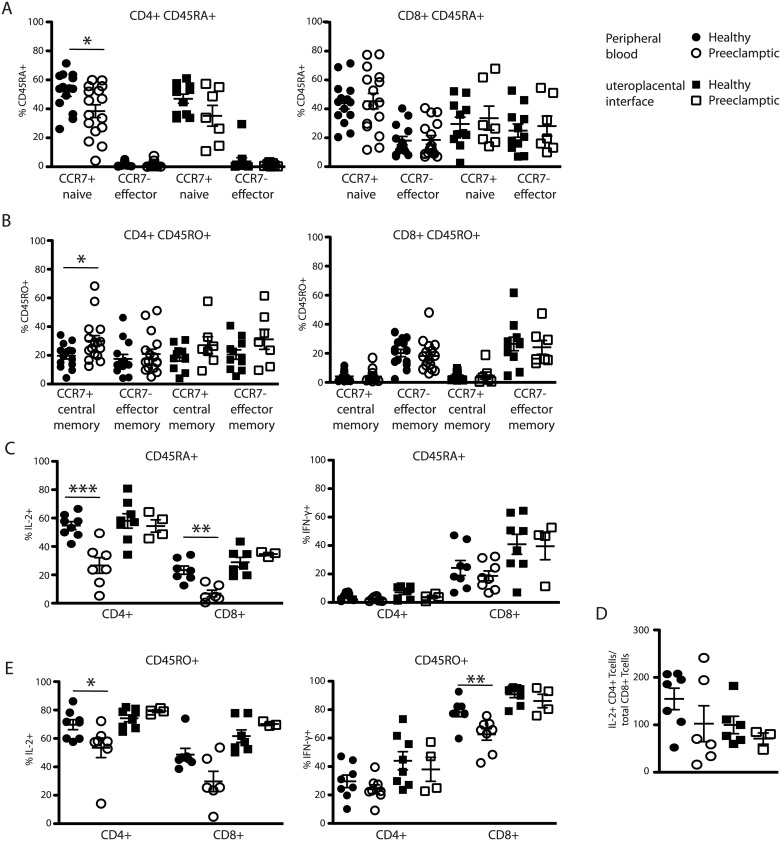
Naïve CD4+ T cells in the peripheral blood of preeclamptic pregnant women are significantly reduced. Fresh peripheral blood and UPI samples from healthy 3^rd^ trimester pregnant women and preeclamptic women were processed and analyzed as described in Materials and Methods. Gated starting with Fig 1B lymphocyte gate and subjected to Fig 1A Steps 2–5. (A, B) CD4+ (left side) or CD8+ (right side), CCR7+ or–, CD45RA+ (A) or CD45RO+ (B) sub-populations were identified as previously described[7]. (C, E) Intracellular cytokine staining of naïve (C) and memory (E) CD4+ and CD8+ T cells. PB (circles) or UPI lymphocytes (squares) were cultured for 4.5h as described in Materials and Methods including PMA/Ionomycin and Golgi Plug treatment. (E) Number of IL-2+ CD4 T cells as determined in C) (both left and right) per CD8+ T cell in the same patient in the PB (circles) and UPI (squares) of healthy (solid) or preeclamptic (open) patients. Student’s T test, unpaired *P<0.05, **P<0.009, ***P<0.0005.

Prolonged inflammatory conditions can impact the adaptive immune response. To address whether the T cell subsets of preeclamptic patients displayed differential distribution or activation compared with healthy patients, we utilized the same sampling- and gating strategy as published previously [7], including samples obtained from the uteroplacental interface (UPI), the uterus locale where the placenta attaches to the uterus and the maternal—and fetal systems meet. Samples were obtained from third trimester healthy patients and preeclamptic patients at time of delivery and processed immediately (<12 hours). We found a significant reduction of naive CD4+ T cells and a concomitant significant increase of CD4+ central memory T cells in the peripheral blood of preeclamptic patients (Fig 1A+1B, left hand sides, Table 5, gating strategy S1A and S1B Fig). While the trend was similar in the lymphocyte population from the UPI, there was no statistically significant difference. Neither the proportions of CD8+ effector memory nor CD8+ central memory T cells displayed any consistent differences between healthy- and preeclamptic pregnant patients (Fig 1B, right hand side, S1 Table). An increased proportion of memory T cells suggested T cell activation, but the activation marker HLA-DR was expressed by similar proportions of cells in both patient populations (S1D & S1E Fig, S2 Table).

**Table 5:** Mean percentage +/- SEM naïve, effector, central memory, and effector memory CD4+ cells gated off CD4+ T cells in PB and at the UPI of healthy and preeclamptic (PE) patients, see also Fig 1A+1C.

	Healthy	Preeclamptic
CD4+CD45RA+CCR7+ (naïve) off total CD4+ PB	52.04 ± 3.49	38.44 ± 3.16
CD4+CD45RA+CCR7+ (naïve) off total CD4+ UPI	46.98 ± 3.16	35.22 ± 7.17
CD4+CD45RA+CCR7- (effector) off total CD4+ PB	2.11 ± 0.48	1.42 ± 0.49
CD4+CD45RA+CCR7- (effector) off total CD4+ UPI	3.69 ± 2.62	1.38 ± 0.44
CD4+CD45RO+CCR7+ (central memory) off total CD4+ PB	19.73 ± 2.32	30.06 ± 3.73
CD4+CD45RO+CCR7+ (central memory) off total CD4+ UPI	18.51 ± 2.52	27.1 ± 5.82
CD4+CD45RO+CCR7- (effector memory) off total CD4+ PB	17.56 ± 3.01	20.88 ± 3.43
CD4+CD45RO+CCR7- (effector memory) off total CD4+ UPI	20.51 ± 3.24	31.15 ± 7.02

### Naïve and memory T cells of preeclamptic patients are functionally impaired

Healthy T cells are capable of different effector functions depending on their roles in the immune system. One of the main functions of CD4+ helper T cells is the production of IL-2 while an important CD8+ T cell effector cytokine is IFN-*γ*. To assess T cell functionality in preeclamptic patients, we applied global stimulation to samples from healthy or preeclamptic patients utilizing PMA/Ionomycin. This approach initiates maximum cytokine production without knowledge of the individual T cell specificity. We found the proportion of both CD4+ and CD8+ naïve T cells (CD45RA+) capable of IL-2 production to be significantly reduced in PBL of preeclamptic patients compared to healthy patients (Fig 1C, left hand side, Table 6, isotype control staining S2B Fig). In the memory (CD45RO+) compartment, we observed a reduced proportion of IL-2 producing cells only in the PBL CD4+ T cell population (Fig 1E, Table 6). Interestingly, IL-2 production of both naïve and memory CD4+ and CD8+ T cells was similar in healthy and preeclamptic patients at the UPI (Fig 1C & 1E, Table 6). Physiologically, CD4+ T cell-derived IL-2 is essential to maintain CD8+ T cell viability and the numerical relationship between these two populations is known to be critical for effector T cell maintenance. The mean number of IL-2+ CD4+ T cells per CD8 T cells in the periphery of healthy pregnant patients was higher, albeit not significantly, than in preeclamptic patients (Fig 1D, Table 7). Memory (CD45RO+) CD8+ T cells displayed a reduced capacity for IFN-γ production in the PBL of preeclamptic patients (Fig 1C & 1E, right hand side, Table 8) but Granzyme A, Perforin A, and TNF-α protein was similar in the two groups (S1F & S1G Fig).

**Table 6:** Mean percentage +/- SEM IL-2+ CD4 or CD8+ T cells gated off total CD4+ or CD8+ T cells in PB and at the UPI of healthy and preeclamptic (PE) patients, see also Fig 1C & 1E.

	Healthy	Preeclamptic
% IL-2+ off CD4+CD45RA+ PB	54.69 ± 2.83	26.58 ± 5.49
% IL-2+ off CD4+CD45RA+ UPI	58.04 ± 5.09	54.45 ± 4.39
% IL-2+ off CD8+CD45RA+ PB	23.31 ± 2.92	6.81 ± 2.36
% IL-2+ off CD8+CD45RA+ UPI	28.89 ± 3.42	34.63 ± 1.09
% IL-2+ off CD4+CD45RO+ PB	69.71 ± 3.53	53.46 ± 6.93
% IL-2+ off CD8+CD45RO+ UPI	74.21 ± 2.38	79.25 ± 0.93
% IL-2+ off CD4+CD45RO+ PB	48.66 ± 4.36	29.75 ± 7.11
% IL-2+ off CD8+CD45RO+ UPI	61.64 ± 4.41	70.17 ± 0.94

**Table 7:** # +/- SEM IL-2+ CD4+ T cells/ total CD8+ T cells in PB and at the UPI of healthy and preeclamptic (PE) patients, see also Fig 1G.

	Healthy	Preeclamptic
#IL-2+CD4+ T cells/ total CD8+ T cells PB	154.8 ± 22.69	102.5 ± 37.74
#IL-2+CD4+ T cells/ total CD8+ T cells UPI	99.73 ± 18.29	71.1 ± 11.79

**Table 8:** Mean percentage +/- SEM IFNγ+ CD4 or CD8+ T cells gated off total CD4+ or CD8+ T cells in PB and at the UPI of healthy and preeclamptic (PE) patients, see also Fig 1F+1I.

	Healthy	Preeclamptic
% IFNγ+ off CD4+CD45RA+ PB	4.29 ± 0.95	2.21 ± 0.52
% IFNγ+ off CD4+CD45RA+ UPI	7.08 ± 1.66	3.79 ± 1.15
% IFNγ+ off CD8+CD45RA+ PB	24.24 ± 5.24	18.69 ± 3.44
% IFNγ+ off CD8+CD45RA+ UPI	40.85 ± 7.02	39.5 ± 9.5
% IFNγ+ off CD4+CD45RO+ PB	29.6 ± 4.4	24.07 ± 3.04
% IFNγ+ off CD4+CD45RO+ UPI	44.06 ± 6.38	38.03 ± 8.48
% IFNγ+ off CD8+CD45RO+ PB	78.31 ± 3.25	62.6 ± 4
% IFNγ+ off CD8+CD45RO+ UPI	90.76 ± 2.28	86.31 ± 4.75

### Naïve and memory T cells of preeclamptic patients do not display classical signs of exhaustion

**Fig 2:**
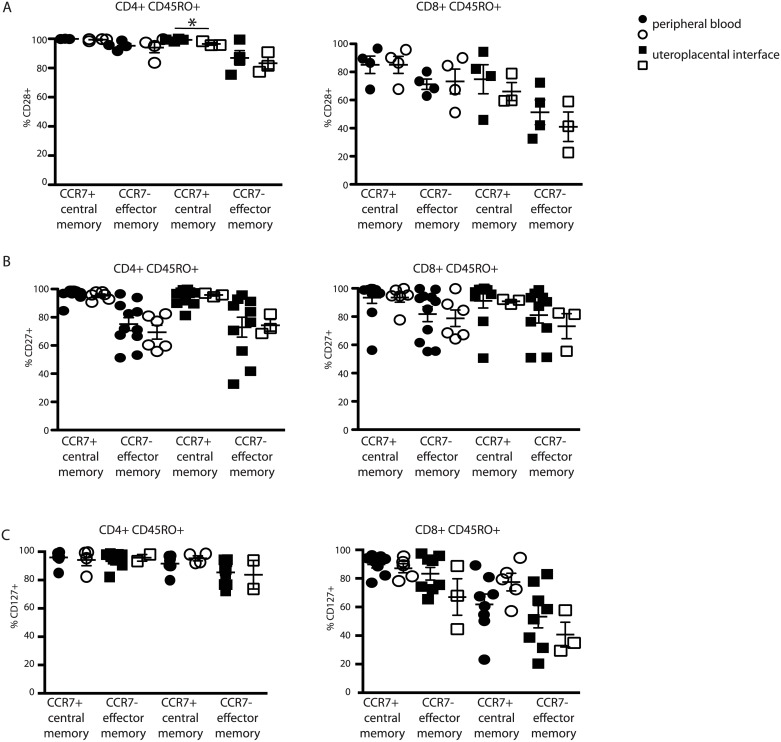
Memory T cells from preeclamptic patients do not display surface markers of exhaustion. Fresh peripheral blood and UPI samples from healthy 3^rd^ trimester pregnant women and preeclamptic women was processed and analyzed as described in materials and methods. CD28 (A) and CD27 (B) percentage of CD4+ memory (left side) or CD8+ memory (right side), CCR7+ or—cells was determined[7]. Student’s T test, unpaired.

Prolonged T cell stimulation can result in exhaustion, a process associated with reduced surface expression of the costimulatory molecules CD27 and CD28 [8]. We analyzed the expression of these two proteins in combination with CCR7 and CD45RO/RA on both CD4+ and CD8+ T cells of healthy and preeclamptic patients. Despite significantly reduced IFN-γ production from decidual CD45RO+ CD8+ T cells in preeclamptic patients, there was no significant difference in CD28 or CD27 expression levels in healthy vs preeclamptic patients (Fig 2A and 2B). Similarly, memory CD4+ T cells from the PBL of preeclamptic patients which are significantly impaired in IL-2 production also did not show any significant differences in CD28-or CD27 expression (Fig 2A and 2B).

### Peripheral activated- and Cytokine-Tregs are significantly expanded during preeclampsia

**Fig 3:**
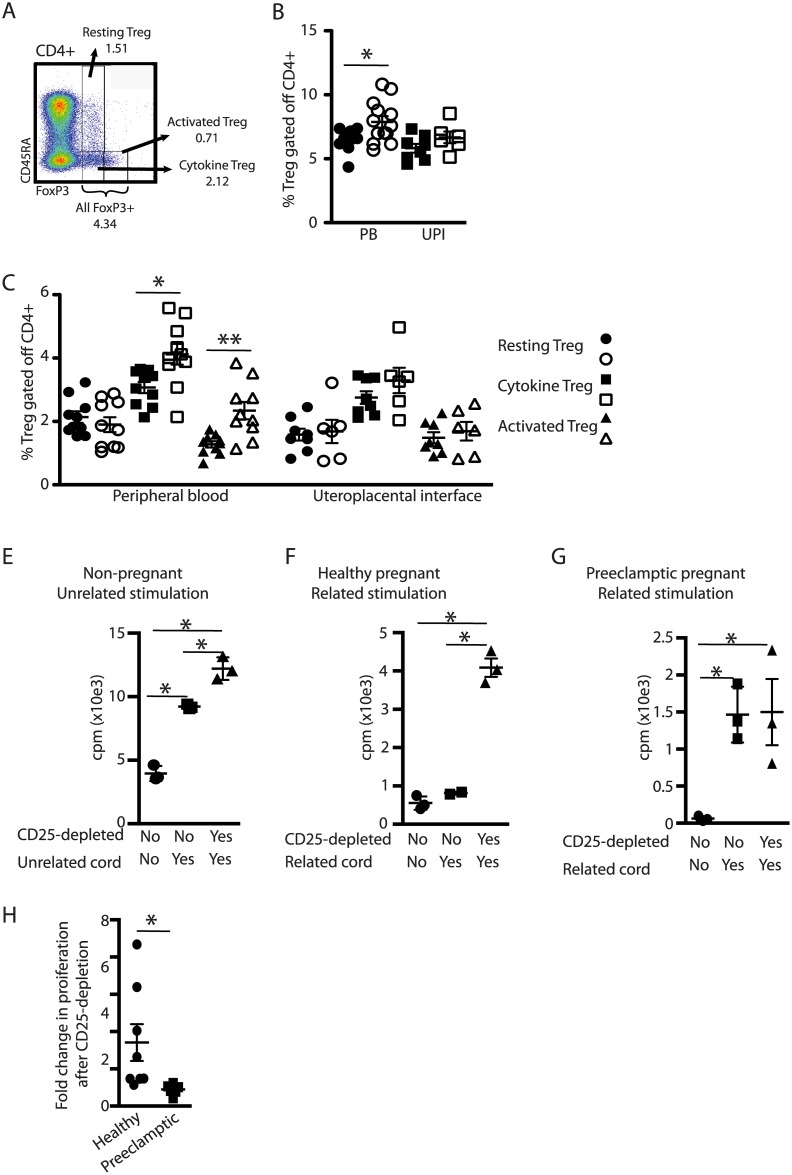
Regulatory T cells of preeclamptic women fail to suppress anti-fetal proliferative responses. (A) Gating strategy for Treg identification. Gating strategy starting with Fig 1B lymphocyte gate and subjecting to S1A Fig Steps until identification of CD4+ T cells. (B) Proportion of total FoxP3+ Treg gated off the total CD4+ populations. (C) % of three Treg subtypes gated off the total CD4+ populations in the PB (left hand side) and UPI (right hand side). (D) Number of CD8+ T cells divided by Cytokine+ Activated Treg as determined in C. (E-G) Proliferative response of maternal PBL to stimulation with related- or unrelated lymphocytes. Donor PBL are exposed to lymphocytes from the indicated sources with (squares) or without (triangles) depletion of CD25+ cells. (E) Donor PBL of a healthy, non-pregnant woman are exposed to unrelated cord blood lymphocytes, (F) PBL of a healthy pregnant woman are exposed to cord blood lymphocytes from her baby and (G) PBL from a preeclamptic pregnant woman are exposed to cord blood lymphocytes from her baby. (H) Fold change in proliferation of healthy (circles) or preeclamptic (squares) pregnant women’s PBL exposed to cord blood lymphocytes of their babies after CD25-depletion. N = 8 normal pregnant women, N = 6 preeclamptic pregnant women total in two separate experiments, representative results are depicted. Statistical analysis in (B, C, D, H) used two-way Student’s T test, unpaired. (E-G) Statistical analysis used one-way ANOVA with Bonferroni post-test. In all test, *p<0.05, **p<0.01.

Regulatory T cells are essential for immune homeostasis of the adaptive-and innate immune system. The most definitive measure of Treg identification is intranuclear FoxP3-staining [9]. Quantification of total FoxP3+ Treg revealed a significant increase of total FoxP3+ CD4+ T cells in the peripheral blood of preeclamptic women (Fig 3A + 3B, Table 9). Previous reports on Treg populations in preeclampsia were heterogeneous possibly due to a lack of Treg subtype considerations as defined by Sakaguchi [10]. In humans, Treg-subtypes are identified by the surface expression of CD45RA in combination with the intranuclear expression of FoxP3 [10]. These three subtypes, Resting Treg (CD45RAhi FoxP3lo), Cytokine Treg (CD45RA- FoxP3lo) and Activated Treg (CD45RA- FoxP3hi) have distinct functional characteristics among which the Activated Treg display the strongest suppression but the lowest proliferation potential [10]. Utilizing this gating strategy, we found significantly more Cytokine Treg and Activated Treg in the PBL of preeclamptic women compared to healthy pregnant women (Fig 3A + 3C, Table 10). In contrast, the proportions of the three sub-populations were comparable at the UPI. Further Treg phenotypic characterization analyzing HLA-DR- expression and CCR6 –expression did not reveal differences between the two populations (S3A & S3B Fig) [11, 12].

**Table 9:** Mean percentage +/- SEM FoxP3+CD4+ T cells gated off total CD4+ T cells in PB and at the UPI of healthy and preeclamptic (PE) patients, see also Fig 1B.

	Healthy	Preeclamptic
% FoxP3+CD4+ T cells gated off total CD4+ T cells PB	6.48 ± 0.29	7.87 ± 0.45
% FoxP3+CD4+ T cells gated off total CD4+ T cells UPI	5.82 ± 0.34	6.67 ± 0.46

**Table 10:** Mean percentage +/- SEM Resting Treg, Cytokine Treg, and Activated T reg gated off total CD4+ T cells in PB and at the UPI of healthy and preeclamptic (PE) patients, see also Fig 3C.

	Healthy	Preeclamptic
% Resting Treg gated off total CD4+ T cells PB	2.14 ± 0.18	1.85 ± 0.19
% Resting Treg gated off total CD4+ T cells UPI	3.07 ± 0.18	3.95 ± 0.29
% Cytokine Treg gated off total CD4+ T cells PB	1.27 ± 0.1	2.14 ± 0.26
% Cytokine Treg gated off total CD4+ T cells UPI	1.58 ± 0.19	1.68 ± 0.37
% Activated Treg gated off total CD4+ T cells PB	2.75 ± 0.19	P<
% Activated Treg gated off total CD4+ T cells UPI	1.48 ± 0.17	1.69 ± 0.29

### Lack of maternal Treg response to fetal antigen during preeclampsia

A hallmark of Treg function is their capacity to suppress the proliferation of effector cells, a feature quantified in proliferation assays. Exposing PBL of healthy, non-pregnant women to irradiated cord-blood of an unrelated source provokes a proliferative response (Fig 3E, squares). This response is mildly enhanced when Treg are depleted via magnetic bead depletion of CD25+ cells (Fig 3E, triangles). Exposure of a healthy pregnant woman’s PBL to the cord blood of her own fetus does not result in any proliferation (Fig 3F, squares) but a proliferative response was detected after Treg depletion (Fig 3F, triangles). In contrast and importantly, exposure of PBL from a preeclamptic woman to the cord blood of her fetus (Fig 3G, squares) provoked a strong proliferative response that was not enhanced further by Treg depletion (Fig 3G, triangles). Comparison of the fold-change in proliferation after CD25-depletion from healthy and preeclamptic women stimulated with the cord of their own babies reveals a significantly increased fold change over the background proliferation (Fig 3H, circles).

## Discussion

Preeclampsia has long been considered as a primarily vascular disorder unique to pregnancy [1]. However, the risk factors for preeclampsia have suggested that an underlying immunopathology is part of the origins of this mysterious, but common disease [13]. The work presented here demonstrates that maternal immune dysregulation is a significant feature of symptomatic preeclampsia. Underlying this immunopathology, we find a significant shift towards a CD4+ memory phenotype with dysfunctional cytokine production by CD4+ and CD8+ T cells in circulation. Unexpectedly, we find an increase in circulating functional Treg subsets, but with lack of function in response to fetal antigen.

In this study, we analyzed the phenotype and functional capacity of T cells from the PB and UPI of healthy and preeclamptic pregnant women at term. Through the combination of surface expression of molecules and intracellular cytokine production in the same cells upon restimulation, we identified a functional exhaustion in combination with population skewing toward memory phenotypes in preeclamptic pregnant women. “classical” surface markers of T cell exhaustion are comparable between healthy and PE pregnant women. Importantly, the phenotype of T cells at term in healthy and PE pregnant women is highly relevant to the goal of understanding the etiology of PE: a T cell’s phenotype may provide information about its antigen encounter, inflammatory environment, and/or kinetics of the preeclamptic condition.

The PB T cell compartment of preeclamptic women displays a significant reduction of naïve T cells and concomitant increase of central memory cells, especially among CD4+ T cells. Transition from naïve (CD45RA) to memory (CD45RO) phenotype requires TCR engagement or high-level unspecific inflammatory stimulation (such as during persistent viral infection). The progression from naïve to memory follows a certain order of events—and kinetics, and has some fundamental differences between mouse and human frustrating the use of animal models for human disease understanding [14–16]. Another factor that increases the complexity of this matter is that the two molecules whose expression is widely used as indicators for T cell exhaustion, CD27 and CD28, are themselves costimulatory molecules and thus are active participants in T cell stimulation and health[17]. The progression of CD8+ T cell differentiation from CD27+CD28+ to CD27+CD28- and lastly CD27-CD28- has been observed repeatedly in human viral infections (Influenza, HCV, EBV, HIV, and CMV), confirmed with telomere length studies and in *in vitro* assays using human cells [18, 19]. According to that classification, the T cell pools from healthy and PE pregnant women largely display the phenotype of Naïve (CD45RA+CD28+CD27+) or Early Activated T cells (CD45RA- or +/- CD28+CD27+) which is as one would expect to find in healthy, young women [19]. In contrast, the CD4+ T cells of PE women who also largely expressed CD27 –and CD28 as one would expect but also expressed CD45RO, a marker thought to identify memory—type T cells. Importantly, this observation was not made for the small subset of fetal—specific maternal cells but the total population of CD4+ T cells. Additionally, memory formation is thought to be irreversible possibly affecting the immune competence of previously PE women for the rest of their lives. In addition to the shift toward memory phenotype, the CD4+ T cells of PE women also displayed a significantly reduced capability of IL-2 which has been observed previously: in people with high-load persistence of HIV infection [16] and in that context, importantly, the low IL-2 production could be rescued by effective drug therapy of HIV [20, 21]. Loss of IL-2 production by CD4 T cells was also observed for persistent, but not resolved, HCV infection [22, 23]. Importantly, those observations were true for antigen-specific T cells, while we observed this reduction of IL-2 production in the total CD4+T cell population. The loss of IL-2 production of these CD4+ T cells is especially puzzling given that they express comparable amounts of CD28 and CD27, the costimulatory molecules whose engagement stimulates IL-2 production in T cells [24, 25]. This indicates disturbances of either the signaling pathways downstream from CD28/CD27 or in the receptor engagement itself respectively in those cells from preeclamptic women.

The reduction in functionality for CD8+ T cells in PE women as indicated by reduced production of IL-2 and IFN-γ, which was unexpected for CD8 T cells with no change in CD45R expression and that, similar to CD4+ T cells express the costimulatory molecules necessary for the induction of cytokine production, CD28-and CD27 in comparable proportions than in healthy pregnant women [26]; this shift was again observed for the total population, not only fetal-specific cells (unless the overwhelming majority of the population of cells are fetal specific; an unlikely situation). Given that the expression of CD27 and CD28 are unchanged and the reduced functionality is true for the whole population, we do not believe that the reduction of functionality is due to a classical exhaustion process as the ones observed in chronic viral infections and cancer. Instead, we observed depressed functionality of the total population, especially production of IL-2 by CD4+ T cells which is essential for maintaining a healthy CD8+ population *in vivo*, as well as thymic induction and peripheral maintenance of Treg functionality [27, 28].

Given that naïve-to memory transition is a natural process that occurs over life and is thought to be irreversible, our observations imply lifelong impacts of preeclampsia on the ability of the adaptive immune system to combat infection [29]. In fact, a reduction of IL-2 production of total lymphocytes in response to global stimulation (in contrast to antigen-specific restimulation as performed in the viral—and tumor studies) was observed when young (23.52+/- 3.47) women were directly compared to older (74.3+/-4.23) women and displayed a significant reduction [30]. Reduced IL-2 production in CD4+ T cells from elderly patients has been studied for quite some time [31–33]. A study by Larbi et al found reduced IL-2 production in CD4+ T cells from elderly patients in conjunction with unchanged expression of CD4 –and CD28, similar to our own observations; again, those patients were considerably older (mean 73 years old) than our own study cohort [34]. Larbi et al demonstrated that changes in lipid rafts in ageing T cells that have a higher cholesterol content in their membranes resulted in lower signal transduction and thus lower activation and IL-2 production [34]. In how far lipid raft changes and/or reduced signal transduction is present in the context of PE is outside of the scope of this paper and awaits investigation.

The question of whether or not the amount of Treg in PB increases or decreases with healthy pregnancy or in preeclampsia has been debated over quite some time now and consensus is not yet on the horizon[35, 36]. In part, even slight differences in experimental design and technique are to blame, the presence or absence of viability stain, time before sample processing, exclusion or not of CD4+CD8+ double positive cells, and lastly whether CD4+ CD25+CD127lo T reg cells are the same population as CD4+FoxP3+ Treg, especially in the context of “non steady-state” such as pregnancy. Attempts for experimental standardization in flow cytometry such as “Standardizing immunophenotyping for the Human Immunology Project” are critical to make different techniques more comparable [37]. In that context, we previously compared the gating strategy approach proposed by the aforementioned group (CD4+ CCR4+CD25hiCD127lo) with intranuclear FoxP3 staining in healthy pregnant women and found that less than 50% of the resting—and cytokine Treg (Resting Treg CD45RA+FoxP3lo, Cytokine Treg CD45RA-FoxP3lo) were identified by that gating strategy [7]. But even if the gating strategies are not identical, we can learn from each other’s data: for example, Steinborn et al conducted a longitudinal examination of different Treg proportions in the PB of healthy pregnant and at term, of pregnant with either PE or HELLP or Preterm Labor necessitating preterm delivery [38]. Even though the gating strategy the authors employ is different from the one used in this study and the authors focused on the relationship of CD45RA and HLA-DR in the FoxP3+ Treg population, the overall pattern the authors observed was similar to our results: CD45RA high Treg (low in FoxP3 and HLA-DR) are reduced in PE compared to healthy pregnant women while CD45RA- Treg (FoxP3/HLA-DR lo and hi) are both increased.

Preeclamptic Treg populations are enriched for the highly suppressive subtypes Cytokine and Activated Treg. At the same time, we find circulating Tregs of preeclamptic mothers unable to regulate maternal lymphocyte proliferative responses to fetal antigens suggesting that these Tregs are either intrinsically deficient or “exhausted”. Since Treg exhaustion is not yet described, we believe that an intrinsic Treg deficiency is part of the disorder. Inability to suppress maternal anti-fetal T cell responses has been observed in preeclamptic women before [35, 36]. Importantly, the design of our experiments does not allow for insights into which interaction partner does not function, the Treg that does not display suppressive properties or the T cell that is resistant to suppression. Both types of dysfunctions have been observed in human diseases: for example, in autoimmune disorders (abnormal recognition of self vs. abnormal recognition of fetus in PE). In autoimmune diseases in which Treg are defective, they transiently shift phenotypes from anti- to pro-inflammatory [39, 40], despite continued expression of the master transcription factor of Tregs, FoxP3. Thus, these patients have bona fide Treg (FoxP3+) that express inflammatory cytokines (e.g., IFNγ). Teff cells resistant to Treg suppression develop this phenotype via many different routes, from TLR1 polymorphisms to improperly activated monocytes, but generally converge on IL-6 perpetuating the resistant state [41–45]. If preeclamptic absence of Treg control mechanistically recapitulates any of those known conditions is subject to future studies.

We observed a significant shift of peripheral CD4 –and CD8- T cells from naïve to memory phenotype as well as significantly reduced functionality in preeclamptic women compared to healthy pregnant women suggesting long-standing immune activation. Importantly, memory formation of T cells is, as far as we understand today, a nonreversible process that is usually driven by time as the major factor. In the case of preeclamptic women, it is unclear what drives this development; the similarly important question, however, pertains to the potential consequences of this change. Are the T cell compartments of previously preeclamptic women permanently “aged”? Does this change in phenotype correspond to permanent changes in functionality and if so, what? How does this change impact the “immunological fitness” of these women at the time of birth and in their subsequent lives?

## Supporting information

S1 Fig Expression of HLA-DR, granzyme A, TNFα, and perforin in the blood and UPI of preeclamptic pregnant women is comparable to healthy pregnant women. Fresh peripheral blood and UPI samples from healthy 3^rd^ trimester pregnant women and preeclamptic women were processed and analyzed as described in Materials and Methods. (A) Gating used to identify single live lymphocytes, CD3+ cells, CD4/CD8+ T cells and CCR7-expression. (B, C) Further sub-populations were identified as previously described[7]. (D, E) HLA-DR-expression and (E, F) intracellular cytokine staining of CD4+ and CD8+ T cells from healthy (solid) or preeclamptic (open) patients’ PB (circles) or UPI lymphocytes (squares) were cultured for 4.5h as described in Materials and Methods including PMA/Ionomycin and Golgi Plug treatment. Student’s T test, unpaired.
(EPS)

S2 Fig Gating strategy and isotype controls for intracellular cytokine experiments. Fresh peripheral blood and UPI samples from healthy 3^rd^ trimester pregnant women and preeclamptic women were processed and analyzed as described in Materials and Methods. (A) Starting with CD4+ or CD8+ live singlets as shown in S1A Fig, CD45RO-expression was used to identify naïve and memory T cells in both the cytokine stained samples and the isotype stained controls. (B) Antibodies against the indicated intracellular cytokines (black line) or the respective isotype control (grey shaded) was quantified in the respective sub-population. Only experiments with isotype control populations <1% were included in the analysis.
(EPS)

S3 Fig HLA-DR and CCR6 are expressed similarly on regulatory T cells of preeclamptic and healthy pregnant women. Expression of HLA-DR (A) and CCR6 (B) on the three Treg subtypes identified as in Fig 3A.
(EPS)

S1 Table Mean percentage +/- SEM naïve, effector, central memory, and effector memory CD8+ cells gated off CD8+ T cells in PB and at the UPI of healthy and preeclamptic (PE) patients, see also Fig 1B+1D. (DOCX)

S2 Table Mean percentage +/- SEM naïve, effector, central memory, and effector memory CD8+ cells gated off CD8+ T cells in PB and at the UPI of healthy and preeclamptic (PE) patients, see also Fig 1B+1D. (DOCX)
