# Supplementary material for: Maternal—Fetal rejection reactions are unconstrained in preeclamptic women
Source: PLoS One. 2017 Nov 27;12(11):e0188250. doi: 10.1371/journal.pone.0188250 (PMC5703473; doi:10.1371/journal.pone.0188250)
Supplement: S1 Table — (DOCX) [file pone.0188250.s004.docx]

|  | Healthy | Preeclamptic |
| --- | --- | --- |
| CD8+CD45RA+CCR7+ (naïve) off total CD8+ PB | 43.97 ± 3.99 | 45.38 ± 5.33 |
| CD8+CD45RA+CCR7+ (naïve) off total CD8+ UPI | 29.57 ± 4.8 | 33.61 ± 8.32 |
| CD8+CD45RA+CCR7- (effector) off total CD8+ PB | 18.12 ± 2.8 | 18.56 ± 3 |
| CD8+CD45RA+CCR7- (effector) off total CD8+ UPI | 24.99 ± 4.56 | 28.1 ± 6.93 |
| CD8+CD45RO+CCR7+ (central memory) off total CD8+ PB | 4.2 ± 0.88 | 4 ± 1.02 |
| CD8+CD45RO+CCR7+ (central memory) off total CD8+ UPI | 4.3 ± 0.91 | 4.65 ± 2.49 |
| CD8+CD45RO+CCR7- (effector memory) off total CD8+ PB | 20.25 ± 2.51 | 18.28 ± 2.61 |
| CD8+CD45RO+CCR7- (effector memory) off total CD8+ UPI | 26.47 ± 4.66 | 24.15 ± 4.74 |

S1 Table
