# Supplementary material for: Maternal—Fetal rejection reactions are unconstrained in preeclamptic women
Source: PLoS One. 2017 Nov 27;12(11):e0188250. doi: 10.1371/journal.pone.0188250 (PMC5703473; doi:10.1371/journal.pone.0188250)
Supplement: S2 Table — (DOCX) [file pone.0188250.s005.docx]

|  | Healthy | Preeclamptic |
| --- | --- | --- |
| % HLA-DR+ off CD4+CD45RA+CCR7+ (naïve) PB | 1.4 ± 0.34 | 1.55 ± 0.32 |
| % HLA-DR+ off CD4+CD45RA+CCR7+ (naïve) UPI | 1.94 ± 0.49 | 1.25 ± 0.34 |
| % HLA-DR+ off CD4+CD45RA+CCR7- (effector) PB | 7.21 ± 1.49 | 9.27 ± 2.79 |
| % HLA-DR+ off CD4+CD45RA+CCR7- (effector) UPI | 5.92 ± 1.64 | 10.95 ± 5.23 |
| % HLA-DR+ off CD4+CD45RO+CCR7+ (central memory) PB | 5.04 ± 0.95 | 7.41 ± 1.67 |
| % HLA-DR+ off CD4+CD45RO+CCR7+ (central memory) UPI | 8.1 ± 2.16 | 6.13 ± 0.96 |
| % HLA-DR+ off CD4+CD45RO+CCR7- (effector memory) PB | 13.31 ± 1.8 | 15.96 ± 2.24 |
| % HLA-DR+ off CD4+CD45RO+CCR7- (effector memory) UPI | 18.47 ± 3.51 | 17.08 ± 3.43 |
| % HLA-DR+ off CD8+CD45RA+CCR7+ (naïve) PB | 7.96 ± 2.45 | 4.67 ± 0.89 |
| % HLA-DR+ off CD8+CD45RA+CCR7+ (naïve) UPI | 6.78 ± 2.26 | 5.41 ± 1.53 |
| % HLA-DR+ off CD8+CD45RA+CCR7- (effector) PB | 20.59 ± 2.05 | 18.74 ± 2.38 |
| % HLA-DR+ off CD8+CD45RA+CCR7- (effector) UPI | 21.65 ± 4.04 | 25.99 ± 4.84 |
| % HLA-DR+ off CD8+CD45RO+CCR7+ (central memory) PB | 19.82 ± 2.49 | 25.3 ± 3.79 |
| % HLA-DR+ off CD8+CD45RO+CCR7+ (central memory) UPI | 26.62 ± 6.7 | 31.49 ± 4.76 |
| % HLA-DR+ off CD8+CD45RO+CCR7- (effector memory) PB | 27.73 ± 3.41 | 28.37 ± 2.69 |
| % HLA-DR+ off CD8+CD45RO+CCR7- (effector memory) UPI | 38.71 ± 6.15 | 41.12 ± 6.34 |

S2 Table
